# Erratum

**DOI:** 10.1002/jgf2.355

**Published:** 2020-07-29

A pagination error occurred in the March 2020 issue (Volume 21, Issue 2) of *Journal of General and Family Medicine*. The first page of the March issue was given the same page number as the first page of the January issue (Volume 21, Issue 1). That is, January page numbers 1–30 were used again in the March issue; however, the March issue contains the correct articles. Only the page numbers were repeated.

## PAGINATION IN MAY ISSUE (VOLUME 21, ISSUE 3)

The March issue ends with page 31, and the May issue begins with page 33, no journal content is missing.

## CITING REFERENCES

When citing references from the March issue in future research and writing, clarify the reference by including the volume number, the issue number, and DOI. These details are necessary for the readers trying to locate correct sources.

The publisher apologizes for the error.

